# The plasma metabolome and clinical features of patients with coeliac disease in Northwest China

**DOI:** 10.1080/07853890.2026.2668756

**Published:** 2026-05-16

**Authors:** Wang Zhang, Xingxing Wu, Yuge Li, Zhenting Huang, Wenjia Hui, Tian Shi, Weimin Lu, Hua Bai, Qi Wu, Mao Wang, Kexin Liu, Luzhou Xu, Feng Gao, Jing Wu

**Affiliations:** ^a^ Department of Traditional Chinese Medicine, Nanjing Drum Tower Hospital, Nanjing Drum Tower Hospital Clinical College of Nanjing University of Chinese Medicine, Nanjing, People’s Republic of China; ^b^ Gastroenterology Department, Henan Province Hospital of Traditional Chinese Medicine, Zhengzhou, People’s Republic of China; ^c^ Gastrointestinal Endoscopy Center, Hezhou Chinese Medicine Hospital, Hezhou, People’s Republic of China; ^d^ Department of Gastroenterology, People’s Hospital of Xinjiang Uygur Autonomous Region, Urumqi, People’s Republic of China; ^e^ Xinjiang Clinical Research Center for Digestive Diseases, Urumqi, People’s Republic of China; ^f^ Internal Medicine Department, Affiliated Hospital of Nanjing University of Chinese Medicine, Nanjing, People’s Republic of China; ^g^ Department of General Internal Medicine, BDA Branch of Dongfang Hospital of Beijing University of Chinese Medicine, Beijing, People’s Republic of China; ^h^ Clinical Research Institute, Affiliated Hospital of Nanjing University of Chinese Medicine, Nanjing, People’s Republic of China; ^i^ Department of Gastroenterology, The 981 Hospital of the Joint Logistics Support Force of the Chinese People’s Liberation Army, Chengde, People’s Republic of China; ^j^ Gastroenterology Department, Affiliated Hospital of Nanjing University of Chinese Medicine, Nanjing, People’s Republic of China; ^k^ The Institute of Chinese Medicine of Nanjing University, Nanjing Drum Tower Hospital, Affiliated Hospital of Medical School, Nanjing University, Nanjing, People’s Republic of China

**Keywords:** Coeliac disease, Chinese population, metabolomics, central carbon metabolism, exposome

## Abstract

**Objective:**

Coeliac disease (CeD) is a relatively common autoimmune disease in Caucasian populations, but is rarely reported in China. The metabolic characteristics of Chinese patients with coeliac disease remain insufficiently characterised. Therefore, this study attempted to describe the metabolic profile of a small cohort Chinese patients with coeliac disease.

**Methods:**

Plasma samples and dietary pattern information were collected from 15 patients with CeD, 15 healthy volunteers, and 30 patients with inflammatory bowel disease (IBD). Plasma metabolomic analysis was performed using a pseudo-targeted metabolomics approach based on ultra-high-performance liquid chromatography coupled with triple quadrupole-linear ion trap mass spectrometry (UHPLC-QTRAP-MS). Dietary intake was assessed *via* food frequency questionnaires, and metabolomics data were adjusted according to energy and 17 specific nutrient covariates.

**Results:**

Despite reporting significantly higher daily intakes of protein and carbohydrate when compared to controls or IBD patients, CeD patients exhibited a relative insufficiency of micronutrient niacin. Several distinct metabolic alterations were identified in CeD patients relative to healthy controls, independent of dietary intake. These included decreased metabolites in the pentose phosphate pathway and increased intermediates of the citric acid cycle. The abundances of L-proline, D-proline, microbiota-related aromatic amino acid metabolites, glycine-conjugated bile acids, and the plant sterol panuosterone were increased. In contrast, the abundances of several long-chain acylcarnitines were reduced despite higher fat intake. These metabolic alterations existed in CeD but not in IBD patients.

**Conclusions:**

CeD patients in Northwest China exhibit a unique metabolic profile distinct from IBD and healthy controls, involving host central carbon metabolism, glycerophospholipid metabolism, gut microbiota, and other factors. This might underscore the multifactorial nature of coeliac disease, implicating other factors beyond genetics and gluten.

## GRAPHICAL ABSTRACT

**Figure:**
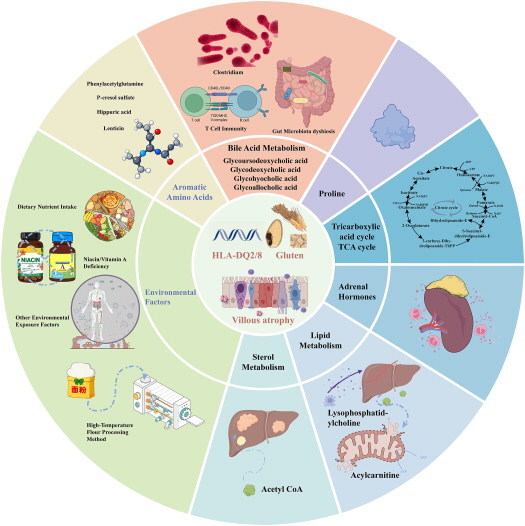

## 1. Introduction

Coeliac disease (CeD) is a T-cell-mediated autoimmune disorder, a specific enteropathy triggered by gluten, affecting ∼1% of the global population [1]. HLA-DQ2 and/or HLA-DQ8 are the major genetic determinants of susceptibility, and a gluten-free diet remains the cornerstone of treatment [2]. However, HLA risk alleles and gluten exposure alone are insufficient to explain disease onset and clinical heterogeneity in CeD [3,4]. Increasing evidence suggests that additional factors, including gut microbiota and viral infections, may also contribute to disease susceptibility and progression [5,6]. In addition, immune-related comorbid conditions such as IgA deficiency have also been discussed in relation to CeD susceptibility [7]. These observations suggest that CeD is associated not only with intestinal immune injury but also with broader systemic alterations. Furthermore, they underscore that its pathogenesis remains to be fully elucidated.

Despite the well-known fundamental pathological mechanisms, major genetic susceptibilities, and effective gluten-free diet therapy, long-term adherence to a gluten-free diet is challenging and may lead to nutritional imbalances, sustaining the need for additional therapeutic strategies. In recent years, several non-dietary therapeutic strategies for coeliac disease have been explored, including anti-gliadin antibodies, transglutaminase 2 inhibitors, enzymatic gluten degradation, and immune-modulating approaches [8–10]. However, these approaches remain under development. Omics-based approaches may help provide a more comprehensive view of CeD, with metabolomics offering a way to characterise associated metabolic features [11,12].

In the last decade, multi-omics approaches have been increasingly applied to the study of CeD, including metagenomic and metabolomic analyses. The proteomic study has suggested that multiple proteins are involved in the pathological changes of CeD [13]. Metabolomic studies have shown that the metabolic pathways of CeD were altered [14,15]. Although findings have not been entirely consistent, previous studies have suggested abnormalities in glycolysis, the Krebs cycle, amino acid metabolism, and lipid metabolism in patients with CeD [16,17]. Microbiome-related studies have suggested that microbial enzymes may influence the hydrolysis of proline-rich gluten peptides, thereby affecting peptide immunogenicity and immune activation in CeD [18,19].

However, these previous findings were derived from relatively limited studies and were not entirely consistent. Moreover, data from Asian populations are scarce. Our preliminary study aimed to characterise pathway-level plasma metabolic alterations in CeD in a Northwest China small cohort using pseudo-targeted metabolomics. Patients with CeD were compared with healthy controls and patients with IBD. Short-term dietary intake was also recorded at the time of sampling.

## 2. Participants and methods

### 2.1. Study design and participants

This was a case-control study including patients with CeD, patients with IBD, and healthy controls. All participants aged 18–75 years were recruited between 19 July 2019 and 1 December 2022. Patients with CeD (*n* = 15) were diagnosed according to the established diagnostic criteria. Patients with IBD (*n* = 30) were also diagnosed according to the corresponding diagnostic criteria. In addition, a group of healthy adult volunteers without evidence of hypertension, diabetes mellitus, or autoimmune diseases was recruited as the healthy controls from the physical examination department (*n* = 15). In the healthy controls, routine blood, urine, and stool examinations, biochemical analyses of liver and kidney function, and electrocardiographic findings showed no significant abnormalities, and their previous medical history was unremarkable.

Participants were excluded if they had infectious colitis, parasitic infection, pregnancy or lactation, or incomplete clinical or laboratory data. Individuals who were considered unsuitable for study participation by the investigators were also excluded.

Written informed consent was obtained from all participants. This study was approved by the Ethics Committee of the Affiliated Hospital of Nanjing University of Chinese Medicine (Approval No. 2019NL-085-02).

### 2.2. Diagnostic criteria

The diagnostic criteria for ulcerative colitis (UC) and Crohn’s disease (CD) were established according to the Consensus Opinions on the Diagnosis and Treatment of Inflammatory Bowel Disease (2018, Beijing) issued by the Inflammatory Bowel Disease Group of the Chinese Society of Gastroenterology, Chinese Medical Association [20]. The diagnostic criteria for coeliac disease were based on the World Gastroenterology Organisation Global Guidelines: Coeliac Disease (2017) [21]. The patients presenting with gastrointestinal symptoms and Anti-Tissue-Transglutaminase (anti-tTG) IgA levels > 20 CU were defined as positive, accompanied by villous atrophy evaluated according to the modified Marsh-Oberhuber classification system. In patients with normal total IgA levels, anti-tTG IgA concentrations were measured using enzyme-linked immunosorbent assays (ELISA), performed in accordance with the manufacturer’s instructions. The test kit was supplied by INOVA Diagnostics Inc. (United States). All patients with IBD or CeD underwent gastrointestinal endoscopy and histological confirmation.

### 2.3. Dietary data collection and conversion calculation of nutritional components

Based on the characteristics of the dietary structure in “Dietary Guidelines for Chinese Residents and the Balanced Diet Pagoda” issued by the Chinese Nutrition Society [22], the foods included in this survey were sorted out according to the “Dietary Pagoda”. After being categorised into ten major food groups, the food intake status of the study subjects was obtained.

Two 24-hour dietary status surveys were conducted respectively. The reference intake standards refer to the “Dietary Reference Intakes (DRIs) for Chinese Residents” issued by the Chinese Nutrition Society [23].

### 2.4. Pseudo-targeted metabolomics detection method

#### 2.4.1. Sample preparation method

Samples were thawed on ice. Exactly 50 μL of plasma sample was transferred into a 1.5 mL EP tube, and the sampling volume was recorded. The tube was labelled with a serial number. The following steps were performed on ice. Then, 200 μL of cold methanol was added (containing 13 internal standards: Carnitine C2:0-d3: 0.08 µg/mL; Carnitine C8:0-d3: 0.05 µg/mL; Carnitine C10:0-d3: 0.05 µg/mL; Carnitine C16:0-d3: 0.075 µg/mL; LPC 19:0: 0.375 µg/mL; FFA C16:0-d3: 1.25 µg/mL; FFA C18:0-d3: 1.25 µg/mL; CDCA-d4: 0.375 µg/mL; CA-d4: 0.925 µg/mL; L-Trp-D5: 2.125 µg/mL; L-Phe-D5: 1.8 µg/mL; SM (12:0): 0.375 µg/mL; Choline-d4: 1.0 µg/mL). The mixture was vortexed for 2 min and allowed to stand at low temperature for 10 min. It was then centrifuged at 14,000 g for 15 min at 4 °C. Next, 200 μL of the supernatant was transferred into a new EP tube, concentrated by low-temperature centrifugation, and stored at −20 °C until use. Before instrumental analysis, the concentrated metabolite extract sample was reconstituted with 100 μL of 20% methanol/water solution. After complete dissolution, the sample was vortexed, centrifuged, and the supernatant was collected for analysis in both positive and negative ion modes.

#### 2.4.2. Quality control samples

The blank sample was 20% methanol/water solution, and the quality control (QC) sample was prepared by mixing a portion of all actual samples.

#### 2.4.3. Screening of ion pair information

The above QC samples were subjected to untargeted metabolomics analysis (see Appendix for the untargeted metabolomics conditions). The One-MAP platform was used, based on a multi-level qualitative annotation system including qualitative annotation of standard compounds, qualitative annotation of the KEGG targeted database, and qualitative annotation of multi-source mixed databases. Finally, the qualitative annotation results at different levels were integrated and analysed to obtain preliminary qualitative results. Ion pairs were screened from the untargeted qualitative data. After obtaining the ion pairs, different collision energies were tested using an AB5500 MASS SPECTROMETER to obtain the most suitable collision energy. Ion pair information was also supplemented into the ion pair list from previously targeted measured compounds (such as the tricarboxylic acid cycle and glycolysis), including the ion pair list, declustering potential, collision energy, etc. According to the ion pair list, chromatographic and mass spectrometric analyses were performed on all samples.

#### 2.4.4. Chromatographic analysis

Positive ion mode: Column: Waters BEH C8 column (1.7 μm, 2.1 × 100 mm, USA); column temperature: 50 °C; sample volume: 5 μL; flow rate: 0.35 mL/min; mobile phase A: 0.1% formic acid/water; mobile phase B: 0.1% formic acid/acetonitrile; gradient elution program: B phase maintained at 5%, 0–1 min; B phase changed from 5% to 100%, 1.1–11 min; B phase maintained at 100%, 11.1–13 min; B phase maintained at 5%, 13.1–15 min.

Negative ion mode: Column: Waters HSS T3 column (1.8 μm, 2.1 × 100 mm, USA); column temperature: 50 °C; volume: 5 μL; flow rate: 0.35 mL/min; mobile phase A: 0.1% formic acid/water; mobile phase B: 0.1% formic acid/acetonitrile; gradient elution program: B phase maintained at 5%, 0–1 min; B phase changed from 5% to 100%, 1.1–11 min; B phase maintained at 100%, 11.1–13 min; B phase maintained at 5%, 13.1–15 min.

#### 2.4.5. Mass spectrometry conditions

Positive ion mode: Mass spectrometry analysis was performed using a Turbo V^™^ ion source in positive ion mode with multiple reaction monitoring. The parameters were set as follows: vacuum gauge: 2.4 × 10^−5 ^Torr; multiple reaction monitoring detection window: 55 s; target scan time: 0.8 s; ion spray voltage: 5500 V; source temperature: 550 °C; curtain gas: 35; ion source gas 1: 60; ion source gas 2: 60; collision-activated dissociation: −2.0; declustering potential: 80 V; entrance potential: 10 V; collision cell exit potential: 13 V; scan speed: 10 Da per second. Both Q1 and Q3 were operated at unit mass resolution.

Negative ion mode: Mass spectrometry analysis was performed using a Turbo V^™^ ion source in negative ion mode with multiple reaction monitoring. The parameters were set as follows: vacuum gauge: 2.5 × 10^−5 ^Torr; multiple reaction monitoring detection window: 90 s; target scan time: 0.8 s; ion spray voltage: 4500 V; source temperature: 500 °C; curtain gas: 35; ion source gas 1: 60; ion source gas 2: 60; collision-activated dissociation: −2.0; declustering potential: −80 V; entrance potential: −10 V; collision cell exit potential: −15 V; scan speed: 10 Da per second. Both Q1 and Q3 were operated at unit mass resolution.

#### 2.4.6. Data acquisition

After the instrument became stable, 1 blank sample was first acquired, followed by 2 QC samples, and then the actual sample analysis was started. Thereafter, 1 QC sample was inserted after every 10 actual samples (for different types of samples, random sampling was performed and inserted for analysis). After all actual sample analyses were completed, 1 QC sample was finally acquired. All measurement data were acquired using Analyst^®^ TF data acquisition software (version: 1.6.3, AB SCIEX INSTRUMENTS, USA).

### 2.5. Statistical analysis

#### 2.5.1. Analysis of clinical characteristics

Statistical analyses were performed using SPSS version 25.0. Normality was assessed using the Shapiro–Wilk test, and homogeneity of variances was assessed using Levene’s test. For clinical data, comparisons among the three groups were performed according to data distribution and variance characteristics. One-way analysis of variance (ANOVA) followed by Bonferroni post hoc testing was used when the assumptions of normality and homogeneity of variances were met. Welch’s ANOVA followed by Tamhane’s T2 test was used when variances were unequal. When the assumptions for parametric analysis were not met, the Kruskal–Wallis test followed by Dunn’s test with Bonferroni correction was applied. Categorical variables were compared using the chi-square test or Fisher’s exact test, as appropriate. All tests were two-sided, and *p* < 0.05 was considered statistically significant (*p < 0.05, **p < 0.01, ***p < 0.001).

#### 2.5.2. Metabolomics data preprocessing and multivariate analysis

For metabolomic data, metabolites with non-zero values in at least 80% of samples in any group were retained for further analysis. Missing values were imputed using the minimum value for each variable before downstream analyses. Principal component analysis (PCA), partial least squares-discriminant analysis (PLS-DA), orthogonal partial least squares-discriminant analysis (OPLS-DA), and heatmap analysis were performed. Pairwise metabolomic comparisons were conducted between CeD and healthy controls and between CeD and IBD controls. PLS-DA was performed using auto-scaling, with a maximum of five latent variables. Leave-one-out cross-validation was used to evaluate model performance, and permutation testing was performed with 10 permutations.

#### 2.5.3. Screening of differential metabolites and ROC analysis

Differential metabolites were identified according to variable importance in projection (VIP), fold change (FC), and p value, using the criteria of PLS-DA_VIP > 1.0, FC > 1.5 or < 2/3, and *p* < 0.05. Differential metabolites with PLS-DA_VIP > 2.0 and AUC > 0.99 were considered potential diagnostic markers. Volcano plots were generated to visualise differential metabolites between groups according to fold change and *p* values. Receiver operating characteristic (ROC) analysis was performed, and the area under the curve (AUC) was calculated to evaluate the discriminative performance of selected metabolites.

#### 2.5.4. KEGG pathway analysis

Metabolic pathway annotation was performed based on the Kyoto Encyclopaedia of Genes and Genomes (KEGG) database. The rich factor was calculated as the ratio of the number of differential metabolites mapped to a given pathway to the total number of metabolites annotated in that pathway.

#### 2.5.5. Linear mixed-effects model adjustment

To adjust for dietary factors, a linear mixed-effects model was fitted using the lme4 package in R. Diet-related variables included energy, protein, fat, carbohydrate, dietary fibre, cholesterol, vitamin A, vitamin B1, vitamin B2, niacin, vitamin C, vitamin E, calcium, sodium, magnesium, iron, zinc, and selenium. The calibrated peak-area matrix obtained after adjustment was then used for subsequent differential metabolite screening and ROC analysis.

## 3. Results

### 3.1. Baseline characteristics of healthy controls, IBD, and CeD patients

Unless otherwise indicated, differences in gender composition, age, body weight, and BMI among the CeD, healthy control, and IBD groups were not statistically significant. Height was significantly lower in the CeD patients than in the IBD patients. The mean and standard deviation of anti-tTG IgA in the 15 coeliac disease patients were 1792 ± 1683 CU. Anaemia was observed in 46.7% of patients with CeD, 26.7% of patients with IBD (Table 1).

**Table 1.** Baseline characteristics of the study participants.

Variable	CON (*n* = 15)	IBD (*n* = 30)	CeD (*n* = 15)
Age (years)	41.13 ± 14.43	46.17 ± 14.57	43.87 ± 7.17
Female sex, *n* (%)	8 (53.33)	15 (50.00)	13 (86.67)
Body weight (kg)	59.30 ± 10.25	59.47 ± 10.73	57.07 ± 13.98
Height (m)	1.67 ± 0.08	1.67 ± 0.08	1.61 ± 0.06*
BMI (kg/m^2^)	21.13 ± 2.40	21.26 ± 3.62	21.94 ± 4.78
Anti-tTG IgA (CU)	N/A	N/A	1792 ± 1683
Anemia, *n* (%)	0	8 (26.67)	7 (46.7)

*Note*. *CeD vs. IBD, *p* < 0.05. Gender: NS among groups (*p* = 0.0505).

### 3.2. Comparison of dietary intake among healthy controls, patients with CeD, or IBD

**Figure 1.**
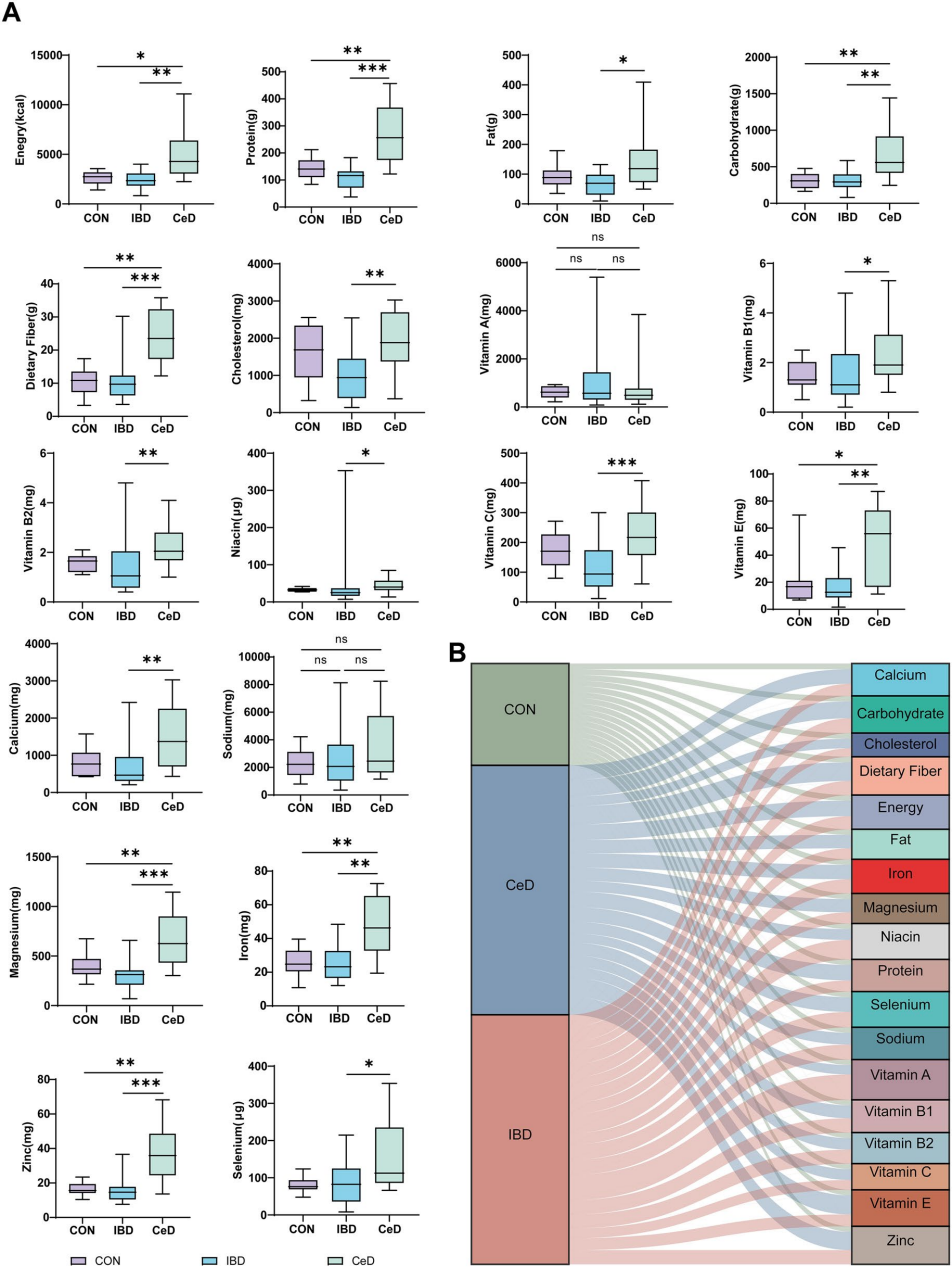
Dietary composition of the three groups. (A) Levels of dietary intake in CON (healthy controls), IBD (inflammatory bowel disease), and CeD (coeliac disease). (B) Sankey diagram showing the dietary composition of CON, CeD, and IBD. *p < 0.05, **p < 0.01, ***p < 0.001.

According to the research results, the intakes of components such as protein, carbohydrates, dietary fibre, vitamin E, iron and zinc in patients with CeD were higher than those in healthy individuals and patients with IBD (*p* < 0.01). However, the intake of niacin is relatively insufficient (Figure 1A,B).

### 3.3. Differential metabolites and potential diagnostic metabolites among groups

The numbers of differential metabolites that simultaneously satisfy VIP > 1.0, FC > 1.5 or < 2/3, and *p <* 0.05 are shown in the following table (Table 2). The full list of differential metabolites is provided in Supplementary Materials 1–6.

**Table 2.** The number of potential differential metabolites in each group.

Group	Number of cationic differential metabolites	Number of anionic differential metabolites
Comparison between CeD and IBD	169	132
Comparison between CeD and Control	200	130
Comparison between IBD and Control	121	80

**Figure 2.**
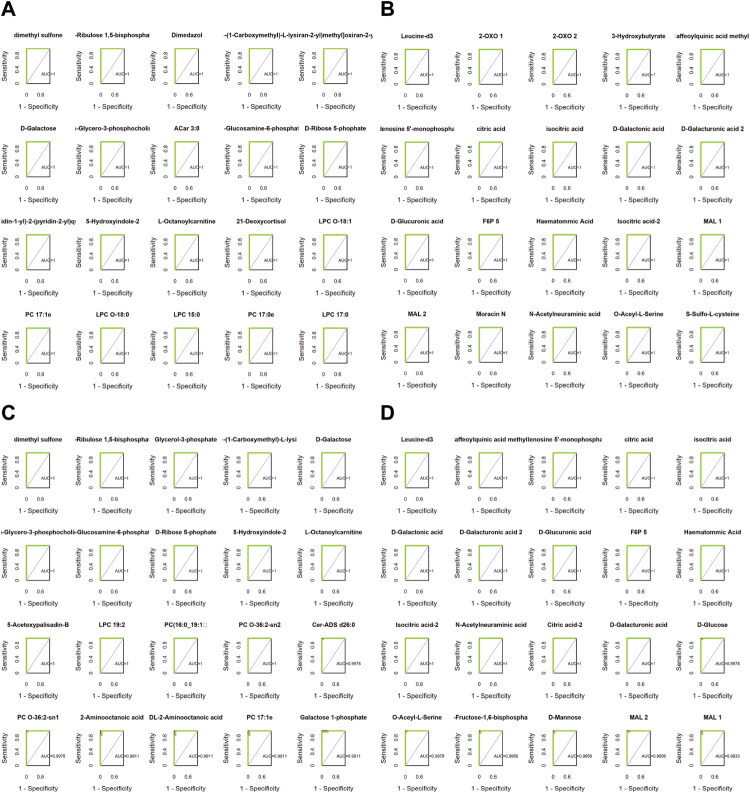
Differential metabolites with AUC > 0.99. (A) Differential metabolites (cations) in CeD vs healthy controls. (B) Differential metabolites (anions) in CeD vs healthy controls. (C) Differential metabolites (cations) in CeD vs IBD. (D) Differential metabolites (anions) in CeD vs IBD.

In addition to meeting the criteria for differential metabolites, metabolites with AUC > 0.99 are presented in Figure 2A–D.

**Figure 3.**
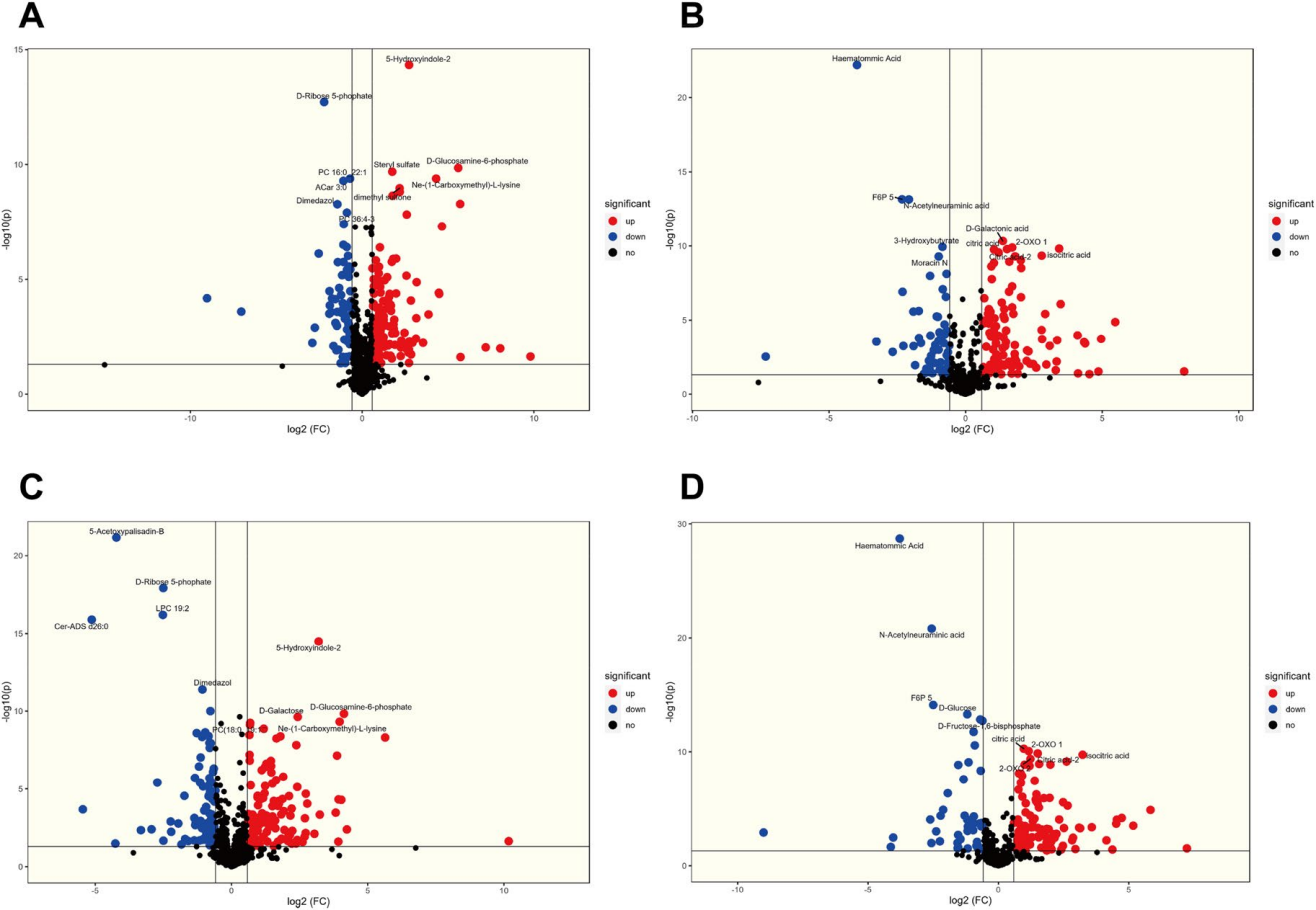
Volcano plots of potential diagnostic metabolites. (A) Volcano plot of cationic potential diagnostic metabolites in CeD vs healthy controls. (B) Volcano plot of anionic potential diagnostic metabolites in CeD vs healthy controls. (C) Volcano plot of cationic potential diagnostic metabolites in CeD vs IBD. (D) Volcano plot of anionic differential metabolites in CeD vs IBD.

Potential diagnostic biomarkers were identified as differential metabolites with PLS-DA_VIP > 2.0 and AUC > 0.99, as described below. Compared with the healthy controls, patients with CeD showed increased abundances of the cationic metabolites N-(1-Carboxymethyl)-L-lysine, dimethyl sulphone, sn-Glycero-3-phosphocholine, D-Galactose, and D-Ribulose-1,5-bisphosphate, whereas Ribose-5-Phosphate, PC 16:0–22:1, Propionyl-L-carnitine, and 2,2′,3′-Epoxyindicolactone decreased (Figure 3A).

Compared with the healthy controls, patients with CeD showed decreased abundances of the anionic metabolites haematommic acid, beta-D-fructose 6-phosphate-2, and N-acetylneuraminic acid, whereas isocitric acid, citric acid-2, 2-ketoglutaric acid-1, D-galactonic acid, and D-galacturonic acid-2 were increased (Figure 3B).

Compared with the IBD group, patients with CeD showed increased abundances of the cationic metabolites N-(1-Carboxymethyl)-L-lysine, D-Galactose, dimethyl sulphone, sn-Glycero-3-phosphocholine, D-Ribulose 1,5-bisphosphate, PC O-36:2-sn2, PC O-36:2-sn1, glycerol-3-phosphate, PC 18:0–19:1, PC 19:1–20:3, LPE 17:0, PC 16:1–19:0, tridecenoylcarnitine, 5-hydroxyindole-2, galactose 1-phosphate, gluconate, ADP, DL-2-aminooctanoic acid-2, LPC 19:1, sphingosine-1-phosphate, PC 33:2, 2-aminooctanoic acid, and 8-hydroxyguanosine, whereas ribose-5-phosphate, 5-acetoxypalisadin B, LPC 19:2, Cer-ADS d26:0, and O-(trans-vaccenoyl)-D-carnitine decreased (Figure 3C).

Compared with the IBD group, patients with CeD showed increased abundances of the anionic metabolites isocitric acid, citric acid-2, D-galacturonic acid-2, D-galacturonic acid, isocitric acid-2, citric acid, ribose-5-phosphate-2, D-glucuronic acid, D-galactonic acid, O-acetyl-L-serine, D-mannose, D-galactose, 5-O-caffeoylquinic acid methyl ester, 2-ketoglutaric acid-1, malate-1, trans-cinnamic acid, 2-ketoglutaric acid-2, D-ribulose 1,5-bisphosphate, and malate-2, whereas beta-D-fructose 6-phosphate-2, haematommic acid, N-acetylneuraminic acid, D-glucose, and D-fructose-1,6-bisphosphate were decreased (Figure 3D).

In comparison between the two groups, the differences in diagnostic markers between CeD and IBD groups mainly lie in the fact that the former shows an increase in multiple carbohydrate metabolites among cations, such as D-Glucose, D-Mannose, D-Galactose, D-Fructose-1,6-bisphosphate, etc., which are involved in Central Carbon Metabolism and related to Glycolysis. There is an increase in multiple lipid metabolites among anions, for example, PC O-36:2-sn2. LPC 19:2, PC O-36:2-sn1, LPE 17:0, as well as Cer-ADS d26:0 and Sphingosine-1-phosphate, are closely related to the metabolism of glycerophospholipids and sphingolipids

### 3.4. Identification of key differential metabolites among CeD patients, IBD patients, and healthy controls

For each pairwise comparison, differential metabolites with OPLS-DA VIP scores > 1 were retained (Supplementary Materials 7–12), as these metabolites were considered to contribute substantially to group discrimination. Their overlap was visualised using a Venn diagram. In the comparison of CeD patients with healthy controls, we identified 29 cationic metabolites (24 up-regulated, 5 down-regulated) and 20 anionic metabolites (15 up-regulated, 5 down-regulated). Similarly, in the comparison between CeD and IBD patients, 27 cationic metabolites (24 up-regulated, 3 down-regulated) and 13 anionic metabolites (11 up-regulated, 2 down-regulated) were significantly altered. In contrast, the metabolic perturbation between IBD patients and healthy controls was less pronounced, with only 18 cationic metabolites (9 up-regulated, 9 down-regulated) and 7 anionic metabolites (2 up-regulated, 5 down-regulated) meeting the selection criteria.

Across both CeD and IBD cohorts, metabolites that differed from healthy controls were primarily enriched in lysophosphatidylcholine (LPC), phosphatidylcholine (PC), bile acids (e.g. glycocholic and deoxycholic acids), acylcarnitines, tricarboxylic acid (TCA) cycle intermediates (particularly succinic acid), and steroid compounds (e.g. dihydrotestosterone sulphate, androsterone sulphate).

**Figure 4.**
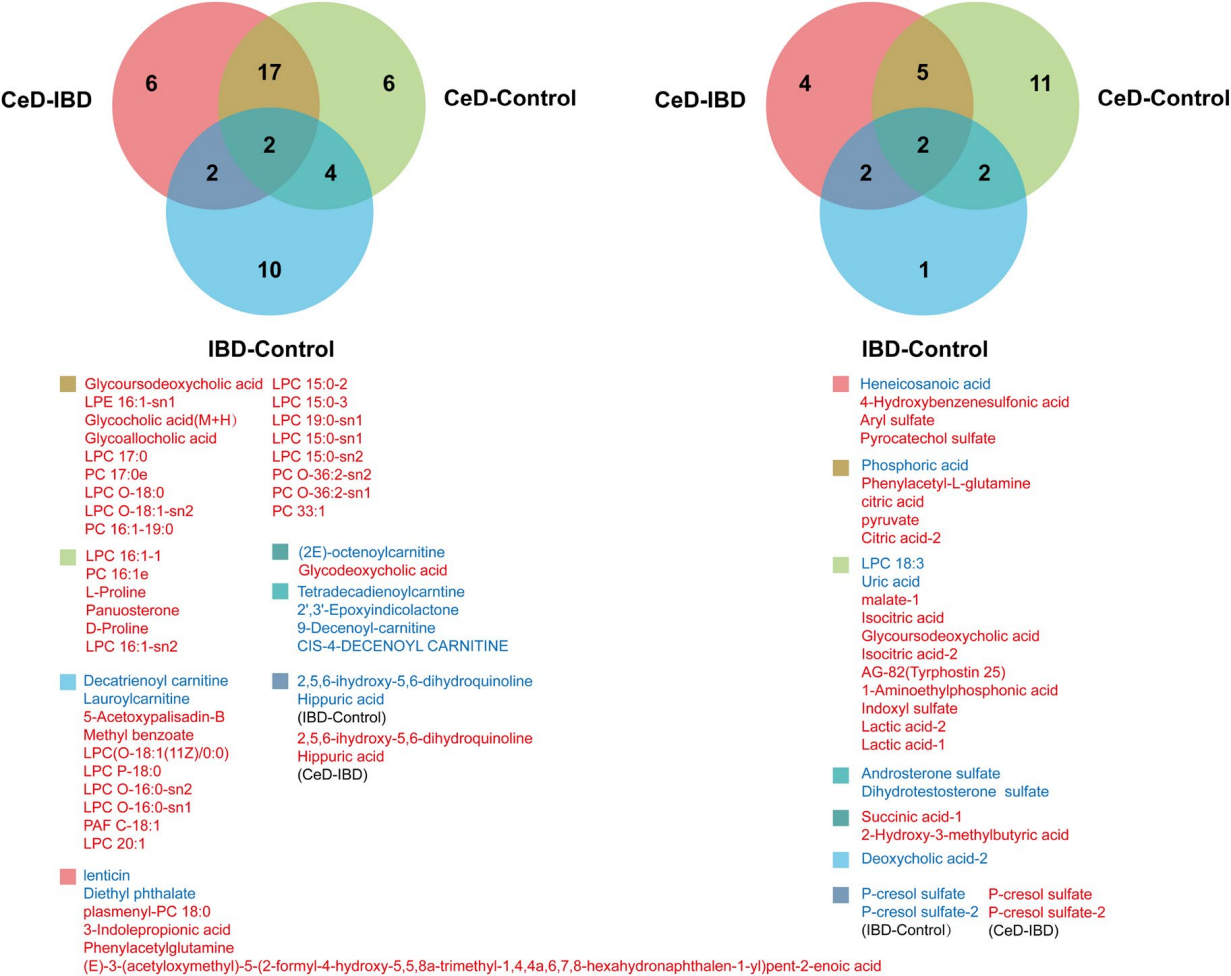
Venn diagram of differential cationic and anionic metabolites with OPLSDA-VIP scores > 1 in CeD vs controls, IBD vs controls, and CeD vs IBD. Red font denotes upregulation; blue font denotes downregulation.

Distinctive patterns emerged in CeD patients. Notably, the cationic metabolite (2E)-octenoylcarnitine was down-regulated, while glycodeoxycholic acid was up-regulated. Anions such as succinic acid and 2-hydroxy-3-methylbutyric acid were markedly increased in the CeD group. A key distinguishing feature of CeD was the concurrent elevation of hippuric acid (a cationic aromatic amino acid metabolite) and p-cresol sulphate (an anionic metabolite), a trend opposite to that observed in IBD patients. Furthermore, CeD patients exhibited significant accumulation of other aromatic amino acid derivatives, including phenylacetylglutamine and indoxyl sulphate, as well as TCA cycle intermediates (isocitric acid, citric acid, pyruvate, and lactic acid). Amino acids closely associated with CeD pathology, such as L-proline and D-proline, were also elevated. Finally, specific steroid alterations were observed in CeD, characterised by an increase in the plant sterol panuosterone. Additionally, the levels of AG-82 (Tyrphostin 25) and 1-aminoethylphosphonic acid were significantly higher in CeD patients (Figure 4).

### 3.5. Differential metabolic pathways in CeD compared with healthy controls and IBD

**Figure 5.**
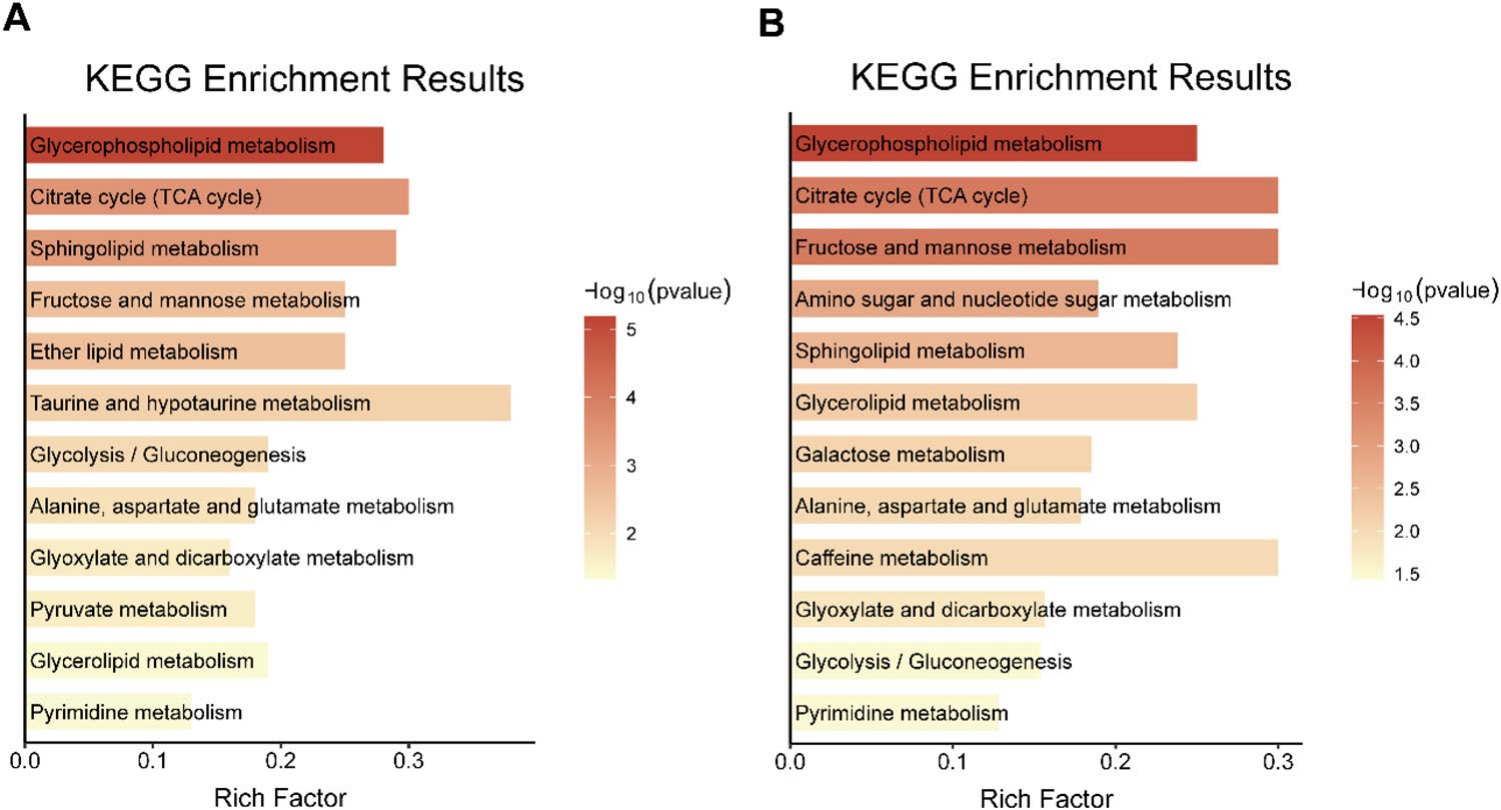
Differentially altered metabolic pathways in patients with CeD compared with healthy controls (A) and IBD patients (B).

At an FDR threshold of <0.05, five metabolic pathways were significantly altered in CeD patients compared with healthy controls: glycerophospholipid metabolism (FDR < 0.001), the citrate cycle, fructose and mannose metabolism, sphingolipid metabolism, and ether lipid metabolism (all FDR < 0.05) (Figure 5A). In the comparison of CeD with IBD patients, five significantly enriched pathways were also identified: glycerophospholipid metabolism (FDR < 0.01), the citrate cycle (FDR < 0.01), fructose and mannose metabolism (FDR < 0.01), amino sugar and nucleotide sugar metabolism (FDR < 0.05), and sphingolipid metabolism (FDR < 0.05) (Figure 5B). Notably, four pathways were common to both comparisons. Ether lipid metabolism was unique to the CeD vs. healthy control comparison, whereas amino sugar and nucleotide sugar metabolism were specific to the CeD vs. IBD comparison.

## 4. Discussion

In the present study, we identified a distinct plasma metabolomic profile in patients with CeD from Northwest China after adjustment for short-term dietary intake. At the pathway level, the main alterations involved decreased pentose phosphate pathway metabolites, increased tricarboxylic acid cycle intermediates, and changes in glycerophospholipid, fructose and mannose, and sphingolipid metabolism. At the metabolite level, L-proline, D-proline, microbiota-related aromatic amino acid metabolites, and glycine-conjugated bile acids were increased, whereas long-chain acylcarnitines were decreased. Disturbances in phosphatidylcholine and lysophosphatidylcholine metabolism were also observed. In addition to intrinsic metabolites, environmentally derived metabolites, e.g. Tyrphostin 25, increased. Several of these alterations differed from those seen in IBD. This study adds to the current literature by describing the plasma metabolome of CeD in a small cohort from Northwest China. It includes both healthy volunteers and IBD patients as controls and takes short-term dietary intake into account.

Central carbon metabolism was significantly altered in CeD. In our cohort, as mentioned before, metabolites associated with the pentose phosphate pathway were decreased, whereas TCA cycle intermediates, including succinic acid, were increased. Succinate, a key TCA intermediate, has been implicated in the pathophysiology of various inflammatory diseases [24–26]. Previous studies in IBD demonstrated increased succinate signalling in the inflamed intestine [27], and recent evidence suggests it may exacerbate gut inflammation by modulating regulatory T cell function [28]. Therefore, the elevated succinic acid observed in CeD likely reflects an inflammatory metabolic state, although its specific mechanistic role in CeD pathogenesis remains to be elucidated.

We observed a coordinated increase in indoxyl sulphate, p-cresol sulphate, phenylacetylglutamine, hippuric acid, and 3-indolepropionic acid in patients with CeD. These metabolites are closely linked to gut microbiota-mediated metabolism of aromatic amino acids and have been implicated in the gut-kidney-heart axis [29,30]. Their collective elevation may represent a distinctive feature of the metabolic profile in this cohort. Notably, the lower levels of some of these metabolites in IBD patients suggest that this pattern is not universal across intestinal diseases. While prospective paediatric studies indicate that gut microbiota and metabolome alterations may precede overt disease onset [31,32], the case-control nature of the present study precludes conclusions regarding causality. Meanwhile, bidirectional Mendelian randomisation based on published Genome-wide association study (GWAS) identified 17 independent SNPs associated with phenylacetylglutamine levels [33], supporting a potential link between host genetics, gut microbiota metabolism, and CeD characteristics.

Our results indicated that plasma glycine-conjugated bile acids, including glycoallocholic acid (GACA), glycoursodeoxycholic acid (GUDCA), glycodeoxycholic acid (GDCA), and glycocholic acid (GCA), were significantly increased in CeD patients compared with healthy controls (Supplementary Material 1 and 2). In contrast, taurine-conjugated bile acids, including TCA and taurodeoxycholic acid (TDCA), were not predominant, a finding consistent with a previous study [15]. Notably, the ratios of GCA/TCA and GDCA/TDCA have been proposed as potential indicators for staging non-alcoholic fatty liver disease and non-alcoholic steatohepatitis [34]. Given these associations, these ratios merit targeted evaluation in future studies assessing hepatic involvement in CeD.

Beyond bile acids, growing evidence points to broader lipid metabolic disturbances in CeD. Pathway analysis in our cohort revealed significant perturbations in glycerophospholipid, sphingolipid, and ether lipid metabolism, with specific alterations in phosphatidylcholine and lysophosphatidylcholine pathways. These findings align with prior nuclear magnetic resonance (NMR)-based studies reporting low serum fat content and persistent abnormalities in lipid classes, including steroids, glycerophospholipids, and fatty acyls, even in patients on a gluten-free diet (GFD) [16]. Specifically, lipid families involved in cell signalling, such as diacylglycerols, lysophospholipids, and ceramides, were dysregulated [17]. Studies have also documented altered choline and choline-derived lipids, particularly phosphatidylcholines [12,35]. When compared with IBD patients, several lipid species (e.g. phosphatidylcholine, ceramide, and sphingosine-1-phosphate) exhibited distinct differences, underscoring lipid dysregulation as a specific component of the CeD metabolomic profile.

Among these lipid alterations, disturbances in acylcarnitine metabolism were particularly notable. Despite the high-fat diet typical of the study region, plasma long-chain acylcarnitine abundance was decreased in CeD patients, suggesting that substrate availability is not the limiting factor. This aligns with previous reports of reduced serum total carnitine in CeD, attributed to small-intestinal malabsorption and transport defects [36,37]. Furthermore, carnitine-ester metabolism was altered even in clinically asymptomatic adults, and GFD may not fully normalise carnitine homeostasis [18]. Serum intestinal fatty acid-binding protein (I-FABP) is a sensitive indicator of epithelial injury [38], and reports have indicated that I-FABP levels do not always normalise after GFD treatment [39,40]. This is further supported by red blood cell fatty acid profiles, which show a pro-inflammatory distribution that is not fully restored after one year of GFD [41,42]. Collectively, the decrease in long-chain acylcarnitine observed in our cohort may be indicative of impaired fatty acid oxidation, linking malabsorption to persistent metabolic dysfunction.

Last but not least, increased levels of L-proline and D-proline were observed in patients with CeD in this study. Interestingly, we previously observed a significant reduction in proline levels in experimental colitis [43], L-proline abundance also decreased in IBD patients in the current study (Supplementary Material 5), suggesting disease-specific alterations in proline metabolism. The abundance of proline residues in immunogenic gluten peptides suggests that alterations in proline metabolism may be relevant to CeD pathogenesis, although the relationship between circulating free proline levels and peptide-bound proline remains unclear [44]. In Northwest China, some wheat-based staple foods, such as nang, are commonly prepared by oven baking, which is a form of high-temperature food processing [45]. A previous study has shown that baking conditions may alter gluten structure and modify the digestion-dependent release profile of coeliac-related immunogenic peptides, including proline-rich peptides such as the immunodominant 33-mer [46]. However, whether such dietary processing characteristics are related to the proline-associated metabolic alterations observed in the present study remains unclear, and this possibility should be regarded as hypothesis-generating only.

Large-sample HLA surveys have suggested that the overall HLA-DQ2/DQ8 susceptibility background in China is lower than that reported in Caucasian populations, although relatively higher frequencies have been observed in northern and northwestern Chinese populations [47]. Thus, genetic susceptibility alone may not fully account for the current recognition pattern of CeD in China. Previous studies have suggested that additional non-genetic factors, including dietary characteristics, food preparation and processing practices, gut microbiota, and other environmental exposures, may also be relevant [48].

Several limitations of the present study should be acknowledged. First, given the case-control design of the study, no conclusions can be drawn regarding temporal relationships or causality. Second, metabolomic profiles may vary depending on nutritional exposure, even among healthy individuals. Although dietary intake was considered in the analysis, such variability may still influence the metabolomic profiles observed in this study. Further studies in larger and well-designed cohorts are needed to confirm the present findings.

## 5. Conclusion

In conclusion, this study identified a characteristic plasma metabolomic pattern in patients with CeD from Northwest China. The observed alterations were mainly related to central carbon metabolism, glycerophospholipid metabolism, and microbiota-related metabolic pathways, with some differences also observed in comparison with IBD. These findings may suggest a multifactorial mechanism in coeliac disease, with factors beyond genetics and gluten potentially contributing to the observed metabolic alterations. Given the case-control nature of the study, these findings should be interpreted as preliminary. Further validation in larger cohorts is required.

## Supplementary Material

Supplemental Material

## Data Availability

The data supporting the findings of this study are available within the article and its Supplementary Materials 1–12. The supplementary materials associated with this manuscript have been deposited in Figshare and are available at https://doi.org/10.6084/m9.figshare.31941843.
